# Evaluating short and long term outcomes following pediatric Myringoplasty with Gelfoam graft for tympanic membrane perforation following ventilation tube insertion

**DOI:** 10.1186/s40463-019-0363-6

**Published:** 2019-08-28

**Authors:** Jin Soo Song, Gerard Corsten, Liane B. Johnson

**Affiliations:** Division of Otolaryngology–Head & Neck Surgery, Department of Surgery, Dalhousie Medical School, Dalhousie University, 5850 College St, Halifax, Nova Scotia B3H 1X5 Canada

**Keywords:** Myringoplasty, Gelfoam, Pediatric, Tympanoplasty

## Abstract

**Background:**

Myringotomy with ventilation tube (VT) insertion to treat recurrent acute otitis media and chronic secretory otitis media has become one of the most common surgical procedures performed in children. Although contemporary literature has detailed the various patient and perioperative factors that affect successful pediatric myringoplasty, there is still limited evidence surrounding the increasing number of graft material options. In particular, gelfoam patching has arisen as a simple and efficient modality for perforation closure, but has a paucity of evidence particularly in pediatric cohorts. Our study aims to evaluate the clinical and audiometric outcomes following gelfoam myringoplasty for TMP following prolonged VT insertion in an urban pediatric population.

**Methods:**

A retrospective review of pediatric patients who underwent myringoplasty between 2013 and 2018 following ventilation tube insertion. Patient demographics, comorbidities, and graft material were correlated with audiometric and clinical outcomes on follow up examination.

**Results:**

One hundred twenty patients underwent myringoplasty, with 61 (50.8%) males with a mean age of 8.9 years old. 101 (84.2%) of patients eventually underwent successful tympanic membrane (TM) closure, with 93 (77.5%) demonstrating closure at initial follow up. In the gelfoam cohort, 77 (90.6%) of patients demonstrated successful TM closure at initial follow up. Overall mean time to closure was 5.6 (standard error (SE) 0.9) months. A multivariate Cox proportional hazards model demonstrated none of the covariates including graft material significantly affected TM closure. Mean change in air conduction threshold were comparable between graft materials.

**Conclusions:**

Pediatric myringoplasty with gelfoam graft material is a safe and viable alternative with favorable short and long term clinical and audiometric outcomes.

## Background

Recurrent acute otitis media and subsequent chronic secretory otitis media are amongst the most common pediatric illnesses [1, 2]. Resultant myringotomy with ventilation tube (VT) insertion, first popularized in 1954 by Armstrong, has become one of the most common surgical procedures performed in children [3, 4]. Complications associated with VT insertion are attributable to consequences arising from chronic otitis media with effusion (COME) treatment and eustachian tube dysfunction, ranging from otorrhea, tube migration, tympanosclerosis, cholesteatoma, and persistent perforation [1, 2, 4]. The majority of tubes spontaneously extrude during postoperative months 6–18 [3, 4], with retained tubes frequently requiring manual removal and subsequent surgical closure. Although the rate of tympanic membrane perforation (TMP) following spontaneous tube extrusion range from 1 to 4%, the rates are significantly increased to 10–28% for retained tubes removed surgically [1, 2].

Myringoplasty was first detailed by Berthold in 1878 following successful closure of a perforation with a full thickness skin graft [5], contemporary techniques entail closure of a TMP without elevating the annulus or entering the middle ear [6]. Variables theorized to attribute to myringoplasty failure rates include tube factors of diameter and duration of placement, to patient parameters of previous adenoidectomy and trisomy 21 status [2, 5]. The current literature surrounding variables for failed myringoplasty are contentious [2, 4, 7]. Furthermore, although most contemporary practice include “freshening the edges” or de-epithelialization to induce inflammatory responses promoting epithelial proliferation [3, 4, 8], selection of the subsequent scaffold material for epithelial migration is still variable.

Several graft materials have been evaluated in an effort to optimize successful outcomes following myringoplasty. Options including hyaluronic acid (HA), autologous fat grafts, tragal cartilage (TC), gelfoam and temporal fascia (TF) have all been proposed for their respective benefits [9, 10]. For example, HA is highly biocompatible due to its natural composition in the extracellular matrix, with physiologic benefits including regeneration and regulation of the fibrous layer with guidance of keratin formation and hyperplastic epithelial tissue during TMP healing [6, 11, 12]. Conversely, fat autografts from dense regions of the abdomen, buttocks and earlobe have been demonstrated to promote cicatrization and revascularization [6, 11, 12].

More recently, gelfoam patching has arisen as a simple and efficient modality for perforation closure, but has a paucity of evidence particularly in pediatric cohorts. Gelfoam is a non-allergenic porous denatured protein sponge with previous trials demonstrating its utility in promoting squamous epithelium migration over an implanted scaffold [13–15]. Our study aims to evaluate the clinical and audiometric outcomes following gelfoam myringoplasty for TMP following prolonged VT insertion in an urban pediatric population.

## Methods

A retrospective chart review of patient records for a group of pediatric patients treated myringoplasty from 2013 to 2018 for persistent TMP following VT insertion. Patients were identified through billing codes for the procedure, with pre-operative diagnosis corroborated with operative reports. Patient exclusion criteria were as follows: 1) over the age of 16 2) concurrent otologic disease diagnosis (e.g. cholestatoma), 3) intraoperative findings of active infection 4) Prior otologic surgery excluding myringotomy with ventilation tube insertion 5) myringoplasty within 24 months of previous ventilation tube insertion and 6) myringoplasty for reasons beyond persistent TMP after tube insertion. All eligible patients during the study period were included. The patients were brought to the operating room suite and under spontaneous respiration with inhalational anaesthetic, the ears were examined with the microscope. In the presence of an indwelling myringotomy tube, the tube was removed with an alligator forceps with a working assumption that this process removes the edge of the tympanic membrane perforation (TMP). If no tube was present, and the perforation was deemed stable, the perforation edge was refreshed via postage stamping with a Schuknecht needle and removed with a cup forceps.

Once the tube is removed or the perforation edge is refreshed, then the gelfoam is flattened and cut into multiple smaller square pieces of variable size to match the perforation, and overlaid onto the perforation to completely cover the perforation edge. Suction is not generally used as the small amount of blood from the refreshed edges absorb into the gelfoam to create a little ‘blood patch’. In the absence of bleeding, the adjacent area of the TM is scratched superficially with the Schuknecht needle to entice a small droplet of blood prior to placing the gelfoam. The gelfoam is not allowed to straddle the hole. No drops are used to prolong patch placement during self-dissolution. Patients are asked to keep the ear dry for 4 weeks. Data was aggregated from the regional electronic database, including patient demographics, comorbidities, operative findings and post-treatment clinical observation and audiometric outcomes during routine follow up. Post-operative appointments occurred at approximately three, nine and 24 months following surgery. Local ethics approval from the IWK-Research Ethics Board was obtained prior to study commencement (File No. 1023345).

Patient information was masked and each individual provided a subject number, compiled in a master list. A separate study database was constructed to incorporate patient age, sex, potential co-morbidities (history of adenoidectomy, trisomy 21 status, cleft lip and/or palate), graft material (gelfoam, HA, fat, TF, TC) intraoperative contralateral ear status (abnormal noted with TMP or COME), retained tube at time of surgery, complications, pre and post-myringoplasty pure tone audiogram (PTA) and speech reception threshold (SRT) values, and clinical evidence of successful closure during follow up examination.

Descriptive statistics was used to detail patient demographics. A multivariate Cox proportional hazards model was used to determine which variables were associated with successful TM closure. Mean change in PTA was calculated by subtracting the mean value from the follow up visit from the mean value preoperatively. Mean PTA and SRT values per graft material were compared within follow-up period using ANOVA analysis. All tests were performed using SPSS (IBM SPSS Statistics 24).

## Results

Overall 120 patients who underwent myringoplasty between 2013 and 2018 were eligible for study inclusion. The mean age of the cohort was 8.9 years old (±10.2). 61 patients were male (50.8%) and 59 were female (49.2%). 62 patients (51.7%) were noted to have retained ventilation tube at the time of surgery. 38 patients (31.7%) had an ‘abnormal contralateral ear’ representing either contralateral TMP or COME. With respect to co-morbidities, four patients (3.3%) had positive Trisomy 21 status, 9 patients (7.5%) had a cleft lip and/or palate, and 23 patients (19.2%) had a previous adenoidectomy. There were zero perioperative complications. Patient demographics can be seen in Table 1.

**Table 1:**
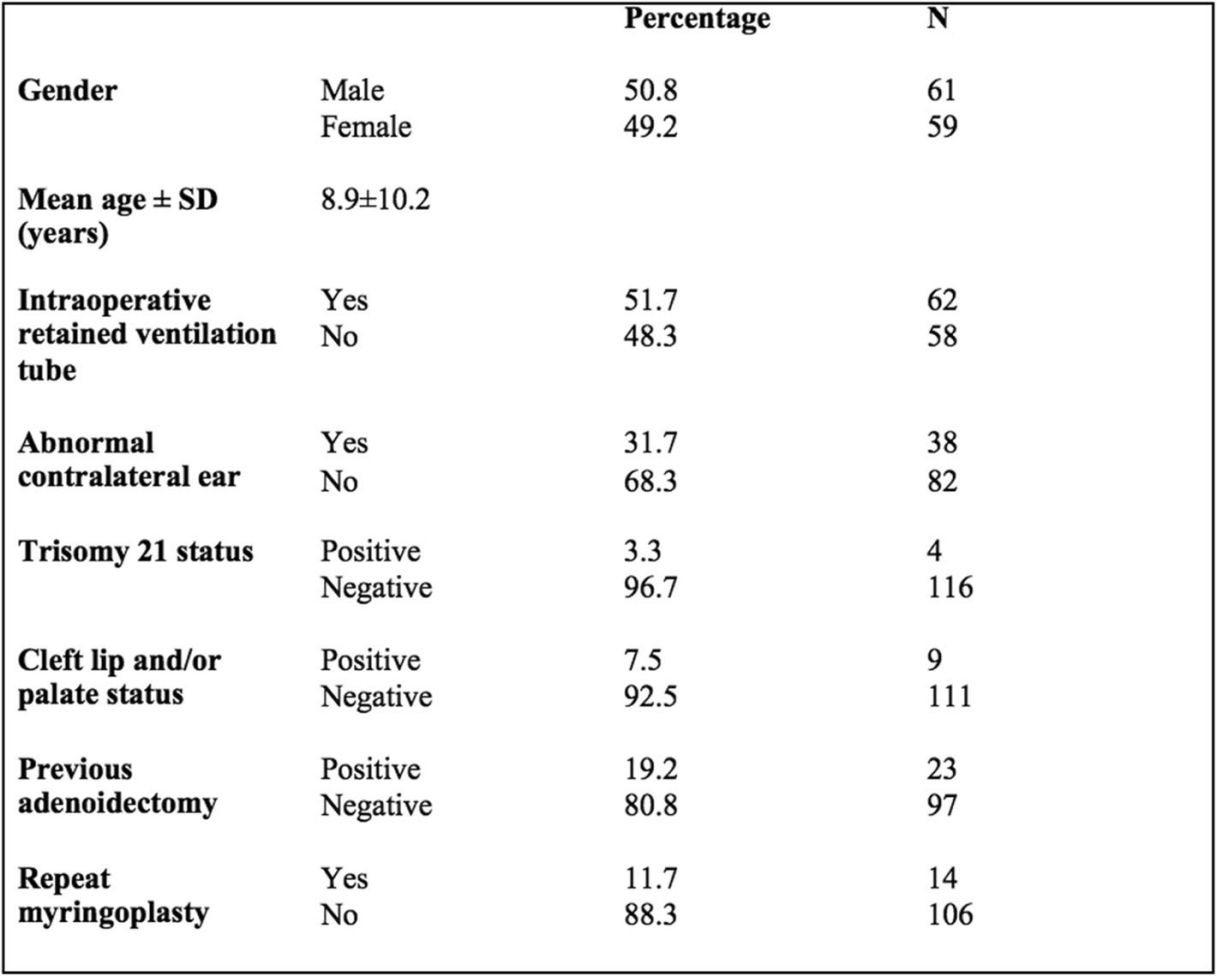
Patient Demographics

**Fig. 1:**
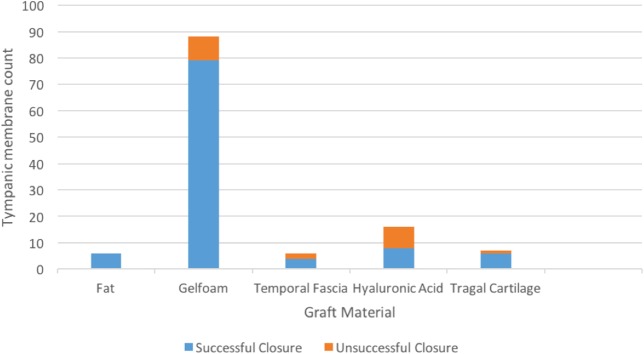
Myringoplasty outcomes for graft materials at initial follow up examination

In total, 101 patients ended up with TM closure (84.2%) following a single myringoplasty procedure. During the follow up period, 93 patients (77.5%) had closure at during the initial follow up examination (Fig. 1), of which four patients subsequently failed. Four further patients (3.3%) had closure at second follow up, and an additional 8 patients (6.7%) had closure by the third follow up visit. Overall, 14 patients (11.7%) had a repeat myringoplasty during the study duration versus a wait and watch approach. Of the 14 patients that had a repeat myringoplasty, 9 (64.3%) demonstrated successful TM closure. Two of the four patients whose graft failed following initial TM closure were included in the repeat myringoplasty treatment arm. The distribution of graft materials and respective rates of successful tympanic membrane closure on follow up examinations are noted in Table 2. Mean time to perforation closure was 5.6 (SE 0.9) months. Mean time to follow up was 4.3 (SE 0.7) months, 11.7 (SE 1.3) months, and 20.4 (SE 2.1) months from initial surgery for first, second, and third visits respectively.

**Table 2:**
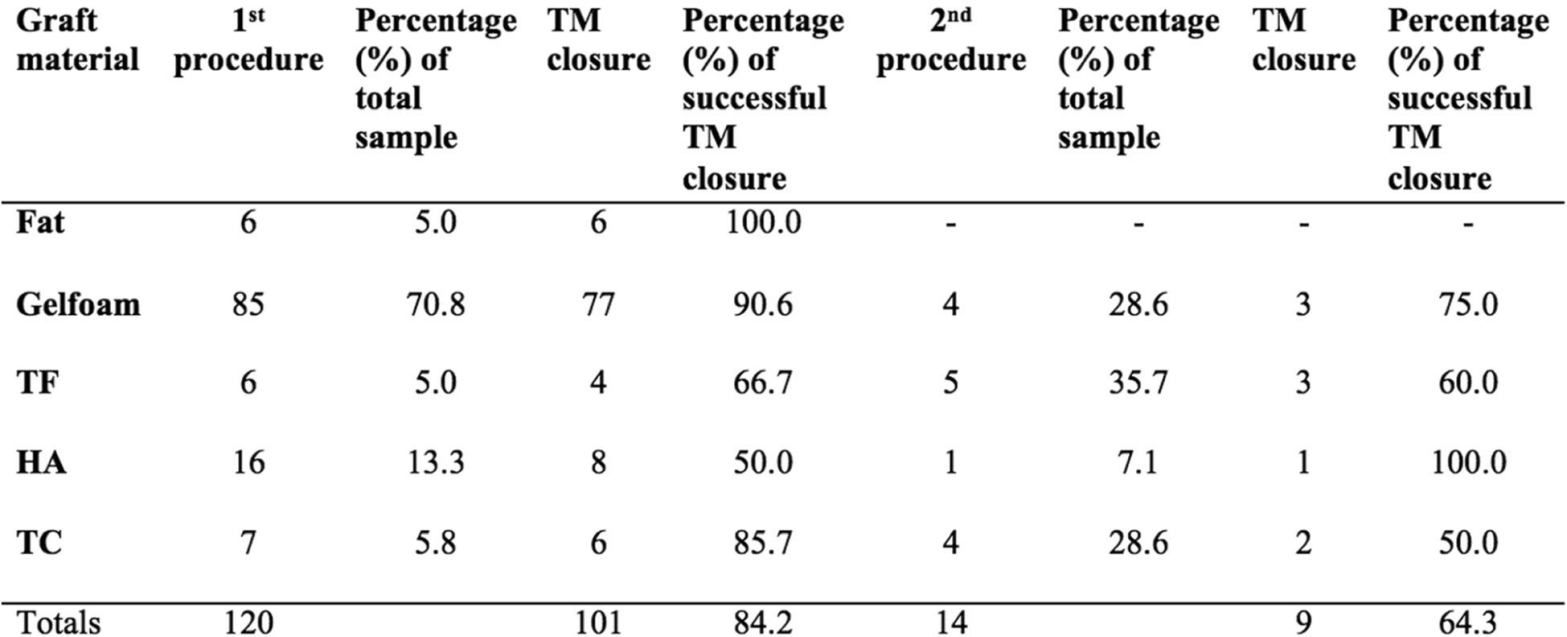
Rates of successful tympanic membrane closure for each graft material group

A multivariate cox proportional hazards model was performed to determine which covariates were independently associated with TM closure. Covariates included: sex, age, indication for myringoplasty, retained tube at surgery, contralateral ear status, graft material, and history of adenoidectomy. None of the six covariates included in the model had a statistically significant effect on TM closure.

**Fig. 2:**
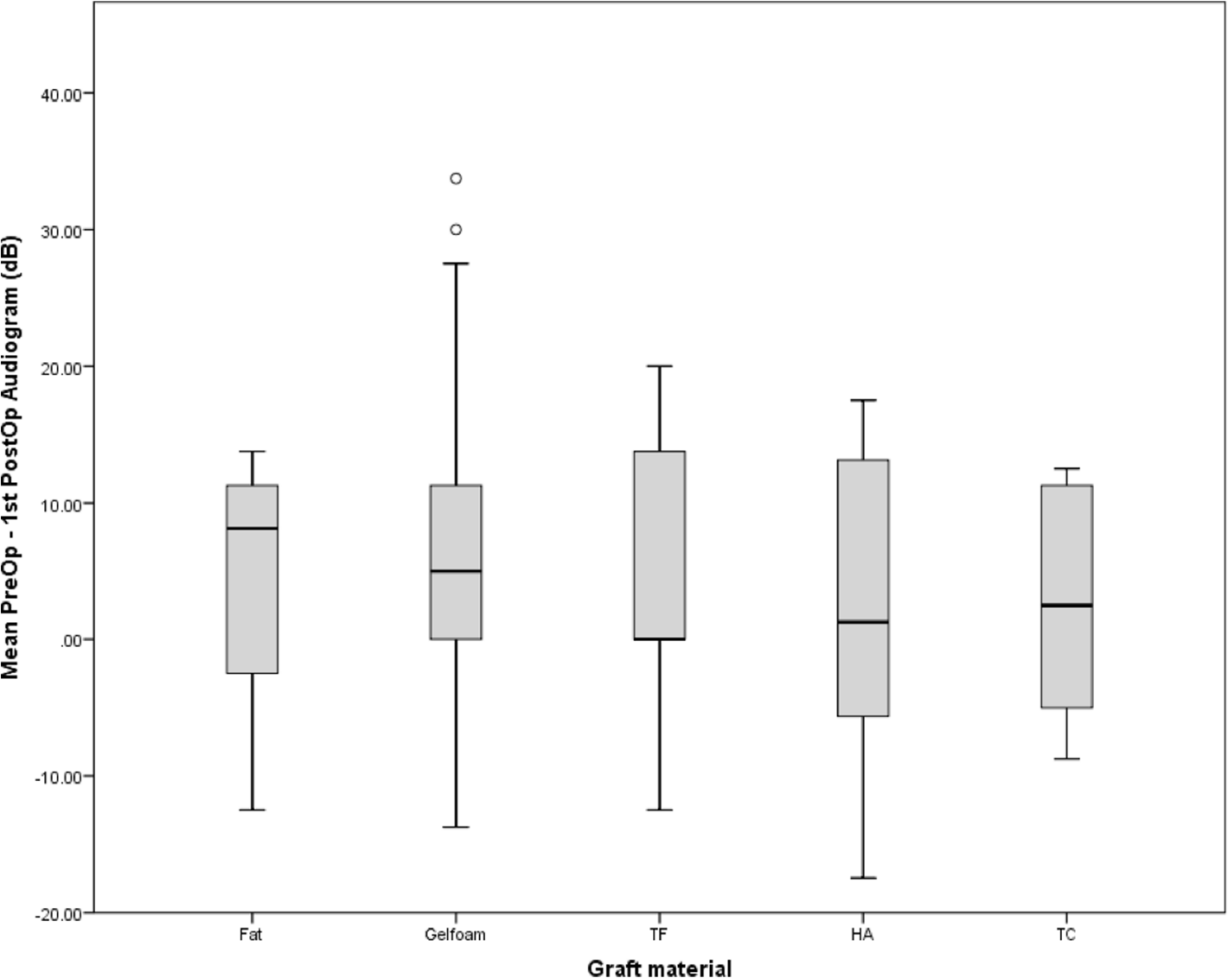
Mean change in PTA between pre and initial post-myringoplasty audiograms for each graft material group

Mean PTA values were compared within each follow-up time point (Table 3). There were no significant differences among the graft materials at any of the time points. Further comparison of mean PTA changes between pre-myringoplasty and initial post-myringoplasty follow up audiograms demonstrated no significant difference across graft material groups (Fig. 2). Mean SRT values were compared within each time point (Table 4). There was a statistically significant difference among the graft materials at the first audiometric follow-up (F (df) = 2.848(4,74), *p* = 0.030), but not at any other time periods.

**Table 3:**
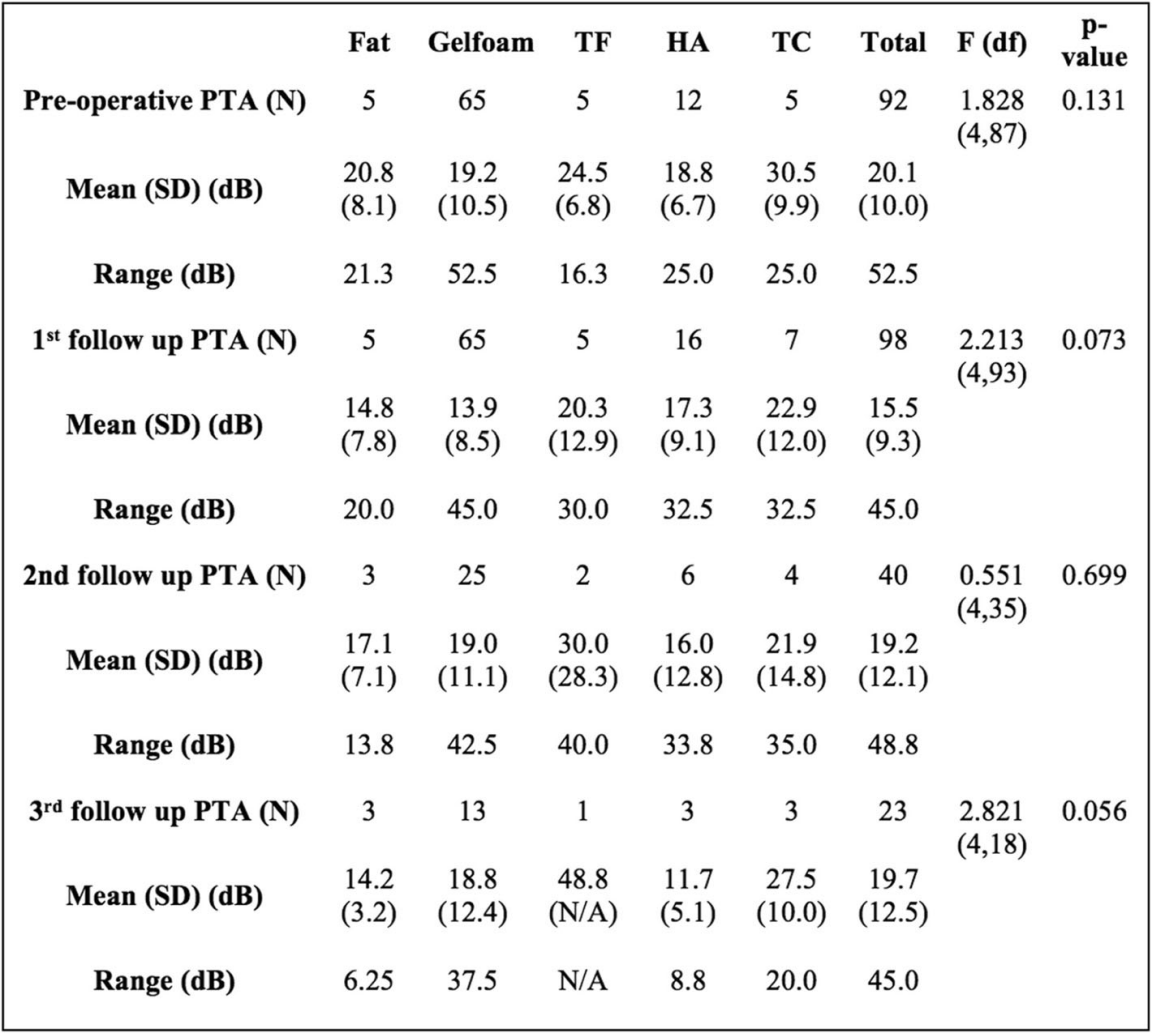
ANOVA analysis comparison of mean PTA for each graft material group within each follow up period

**Table 4:**
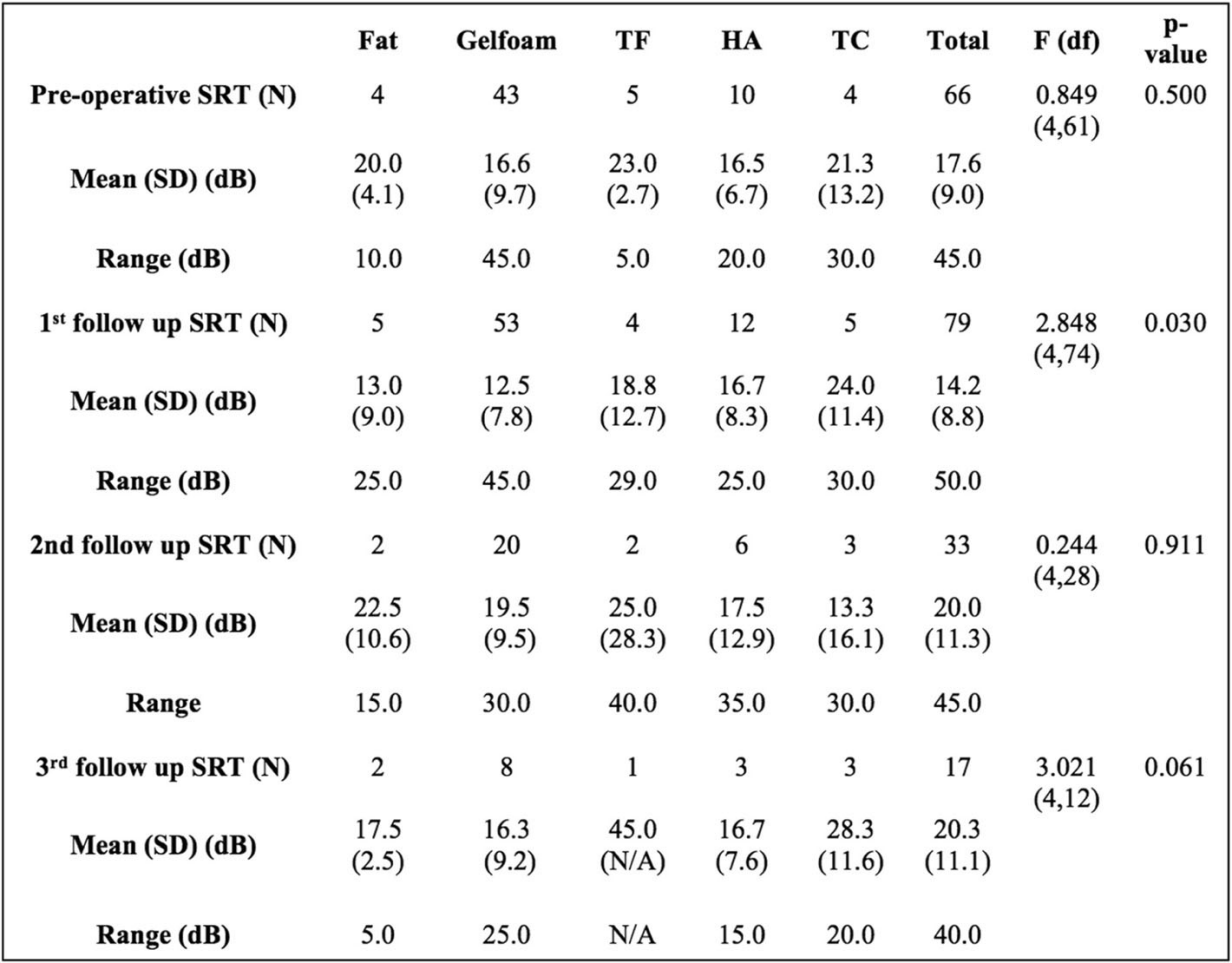
ANOVA analysis comparison of mean SRT for each graft material group within each follow up period

## Discussion

VT insertion is amongst the most common procedures in pediatric otolaryngology, with resultant TMP following prolonged tube placement also occurring with relatively high frequency. The existing literature surrounding graft material within pediatric myringoplasty is outdated with little investigation of more recent graft options. Specifically, although gelfoam has arisen as a promising alternative, there is minimal literature evaluating it’s utility in pediatric myringoplasty.

Regarding parameters influencing surgical outcomes, our results are in keeping with previous studies depicting the majority of variables do not cause a significant effect on outcomes [4, 7, 16, 17]. A large retrospective study of 341 pediatric cases found no difference in TMP rates by patient sex, indication for tube removal, required number of set of tubes or duration of tube retention [4]. Similarly, a tertiary care study of adult patients with COME found that amongst the various risk factors evaluated, contralateral ear status was the sole variable to play a significant role, with worse prognosis for successful uptake when contralateral COME was present [18]. This was corroborated by a recent meta-analysis of 32 studies involving pediatric myringoplasty by Hardman and colleagues, concluding larger perforations or abnormal contralateral ear status were the only variables significantly associated with lower TMP closure rates [7].

Our study population outlined in Table 1 are representative of the expectant patient demographics [7]. Our overall successful perforation closure rate of 84.2% following initial myringoplasty is comparable to the cited literature values [6, 7, 16]. The aforementioned meta-analysis by Hardman et al. found an overall mean weighted closure rate of 83.4% [7]. Our mean time to perforation closure is reflective of the literature describing near definitive outcomes by six months post-myringoplasty [19].

With respect to success rates with specific graft material options, the existing literature cites a broad range of outcomes. A mixed technique study by Schraff et al. of 95 pediatric patients found comparable rates of fat graft versus gelatin film myringoplasty at 94 and 92% respectively [3]. Conversely, a retrospective study by Gun et al. evaluating successful TMP closure between fat graft, HA and temporalis fascia found comparable results between the three treatment groups [11]. Singh et al. cited similar rates in their 220 cases incorporating TF, TC and fat graft myringoplasty to yield successful TMP closure in 95, 90 and 90% of cases respectively [10]. A study by Sanchez et al. evaluating 142 pediatric myringoplasty cases by a single primary surgeon using either temporalis fascia, rice paper, or perichondrium graft material yielded a 74.6% overall successful TMP closure rate [19]. Lastly, a study by Knutsson et al. found that with fat graft myringoplasty 83% of pediatric cases demonstrated successful perforation closure at initial follow up examination, with a PTA of 16.6 dB at one year post-myringoplasty [16]. The lower myringoplasty success rates in our TF and TC groups are attributable to sampling error from a smaller sample size in these respective groups, as the predominant graft material at our institution is gelfoam.

When evaluating gelfoam material specifically, a prospective study of pediatric and adult patients with 2-4 mm sized TMP found an 83% successful closure rate with gelfoam myringoplasty with a PTA change from 19 to 16 dB postoperatively [9]. Likewise, a larger scale tertiary care retrospective study found in 604 children who underwent myringoplasty, gelfoam myringoplasty was the most successful with a 90.8% successful TMP closure rate [19]. Hence our results are congruent with existing literature demonstrating the favorable outcomes with this relatively straightforward technique.

Our results showed favorable rates of TMP closure with gelfoam myringoplasty upon post-operative clinical examination. Furthermore, the audiometric results consolidate comparable results with other graft materials, as both PTA and SRT become similar across groups upon long term assessment. Hence, relative to more cumbersome techniques, including fat, TC and TF which introduce the additional complexity and risks associated with donor site harvesting, gelfoam is a useful alternative with promising outcomes.

The limitations of this study include the sample size imbalance amongst the various graft materials. However, the main goal of the study is to highlight the favorable outcomes with gelfoam myringoplasty, as other techniques have been adequately evaluated in previous studies. Secondly, limitations include the retrospective nature and the inherent variability with multiple primary surgeons. However, as our patient demographics are representative of the broader population and previous studies have illustrated the lack of significance of various patient co-morbidities, we feel this does not markedly impact our ability to make meaningful conclusions. Furthermore, similar to the predilection to use gelfoam for graft material, the high degree of procedural homogeneity at our institutional site restricts technical variability amongst surgeons.

## Conclusion

Conventional approaches to persistent TMP following VT extrusion in pediatric patients has primarily entailed donor site harvesting for various graft materials. The present study suggests a gelfoam myringoplasty is a viable alternative with comparable short and long term clinical and audiometric outcomes.

## Data Availability

The datasets used and/or analysed during the current study are available from the corresponding author on reasonable request.

## Abbreviations

COME: Chronic otitis media with effusion
HA: Hyaluronic acid
PTA: Pure tone audiogram
SRT: Speech reception threshold
TC: Tragal cartilage
TF: Temporal fascia
TMP: Tympanic membrane perforation
VT: Ventilation tube

## References

[1] Nichols PT, Ramadan HH, Wax MK, Santrock RD (1998). Relationship between tympanic membrane perforations and retained ventilation tubes. Arch Otolaryngol Head Neck Surg..

[2] Puterman M, Leiberman A (2005). Gelfoam plug tympanoplasty concomitant with removal of retained ventilation tubes. Int J Pediatr Otorhinolaryngol.

[3] Schraff SA, Markham J, Welch C, Darrow DH, Derkay CS (2006). Outcomes in children with perforated tympanic membranes after tympanostomy tube placement: results using a pilot treatment algorithm. Am J Otolaryngol.

[4] Vercillo NC, Xie L, Agrawal N, Nardone HC (2015). Pediatric Tympanostomy tube removal technique and effect on rate of persistent tympanic membrane perforation. JAMA Otolaryngol-- Head Neck Surg.

[5] Das A, Sen B, Ghosh D, Sengupta A (2015). Myringoplasty: impact of size and site of perforation on the success rate. Indian J Otolaryngol Head Neck Surg Off Publ Assoc Otolaryngol India.

[6] Alzahrani M, Saliba I (2015). Hyaluronic acid fat graft myringoplasty vs fat patch fat graft myringoplasty. Eur Arch Oto-Rhino-Laryngol Off J Eur Fed Oto-Rhino-Laryngol Soc EUFOS Affil Ger Soc Oto-Rhino-Laryngol - Head Neck Surg.

[7] Hardman J, Muzaffar J, Nankivell P, Coulson C (2015). Tympanoplasty for chronic tympanic membrane perforation in children: systematic review and meta-analysis. Otol Neurotol Off Publ Am Otol Soc Am Neurotol Soc Eur Acad Otol Neurotol.

[8] Park S-N, Kim H-M, Jin K-S, Maeng J-H, Yeo S-W, Park S-Y (2015). Predictors for outcome of paper patch myringoplasty in patients with chronic tympanic membrane perforations. Eur Arch Oto-Rhino-Laryngol Off J Eur Fed Oto-Rhino-Laryngol Soc EUFOS Affil Ger Soc Oto-Rhino-Laryngol - Head Neck Surg..

[9] Niklasson A, Tano K (2011). The Gelfoam® plug: an alternative treatment for small eardrum perforations. Laryngoscope.

[10] Singh BJ, Sengupta A, Das SK, Ghosh D, Basak B (2009). A comparative study of different graft materials used in myringoplasty. Indian J Otolaryngol Head Neck Surg Off Publ Assoc Otolaryngol India..

[11] Gün T, Boztepe OF, Atan D, İkincioğulları A, Dere H (2016). Comparison of hyaluronic acid fat graft Myringoplasty, fat graft Myringoplasty and temporal fascia techniques for the closure of different sizes and sites of tympanic membrane perforations. J Int Adv Otol.

[12] Saliba I, Knapik M, Froehlich P, Abela A (2012). Advantages of hyaluronic acid fat graft myringoplasty over fat graft myringoplasty. Arch Otolaryngol Head Neck Surg.

[13] Baldwin RL, Loftin L (1992). Gelfilm myringoplasty: a technique for residual perforations. Laryngoscope.

[14] Park AH, Hughes CW, Jackson A (2006). Crosslinked hydrogels for tympanic membrane repair. Otolaryngol--Head Neck Surg Off J Am Acad Otolaryngol-Head Neck Surg.

[15] Lou Z-C, He J-G (2011). A randomised controlled trial comparing spontaneous healing, gelfoam patching and edge-approximation plus gelfoam patching in traumatic tympanic membrane perforation with inverted or everted edges. Clin Otolaryngol Off J ENT-UK Off J Neth Soc Oto-Rhino-Laryngol Cervico-Facial Surg.

[16] Sckolnick JS, Mantle B, Li J, Chi DH (2008). Pediatric myringoplasty: factors that affect success-a retrospective study. Laryngoscope.

[17] Dangol K, Shrivastav RP (2017). Study of various prognostic factors affecting successful Myringoplasty in a tertiary care Centre. Int Arch Otorhinolaryngol.

[18] Knutsson J, Kahlin A, von Unge M (2017). Clinical and audiological short-term and long-term outcomes of fat graft myringoplasty. Acta Otolaryngol (Stockh)..

[19] Sánchez Barrueco A, Lora Pablos D, Villafruela Sanz M, Almodóvar Álvarez C (2015). Pediatric myringoplasty: prognostic factors in surgical outcome and hearing threshold recovery. Acta Otolaryngol (Stockh).

